# Immobilization of Microbial Transglutaminase for the Production of Homogeneous Immunotherapeutic Antibody–Drug Conjugates

**DOI:** 10.32607/actanaturae.27926

**Published:** 2026

**Authors:** M. N. Baranova, K. A. Sapozhnikova, E. L. Gulyak, Y. A. Prokopenko, O. A. Komarova, Yu. A. Mokrushina, I. V. Smirnov, V. A. Korshun, S. S. Terekhov

**Affiliations:** Shemyakin–Ovchinnikov Institute of Bioorganic Chemistry of the Russian Academy of Sciences, Moscow, 117997 Russia; Higher Chemical College of the Russian Academy of Sciences, D. Mendeleev University of Chemical Technology of Russia, Moscow, 125047 Russia; Endocrinology Research Center, Ministry of Health of the Russian Federation, Moscow, 117036 Russia; Faculty of Chemistry, Lomonosov Moscow State University, Moscow, 119991 Russia

**Keywords:** antibody–drug conjugate (ADC), enzyme immobilization, microbial transglutaminase (mTGase), stoichiometrically homogeneous bioconjugates

## Abstract

Antibody–drug conjugates (ADCs) have revolutionized chemotherapy, allowing it to shift from systemic cytotoxic chemotherapy to specific targeting of cells expressing tumor antigens. ADCs thus offer increased therapeutic efficacy and lower toxicity compared to conventional chemotherapy. Bioconjugation of a highly active therapeutic agent to an antibody is one of the key steps in producing ADCs. The most advanced ADC technologies are based on the production of stoichiometrically homogeneous bioconjugates. Enzymatic modification of antibodies using microbial transglutaminase (mTGase) enables the production of biopharmaceuticals with standardized pharmacokinetic, physicochemical, and therapeutic properties. However, side reactions catalyzed by free mTGase in solution reduce the yield and purity of ADCs. In this study, we evaluated the efficiency of various approaches to mTGase immobilization: streptavidin–biotin interaction, the use of an epoxy-activated polymer, and self-immobilization of mTGase. The highest bioconjugation activity was observed when using streptavidin-modified magnetic particles and biotinylated mTGase. However, mTGase self-immobilization on AH-Sepharose 4B or immobilization using epoxy-activated agarose ensures comparable efficiency in enzymatic antibody modification and is the most rational route from the perspective of process scalability. We believe that the proposed approaches are of high practical significance, opening up avenues for optimizing enzymatic bioconjugation and scaling up the production of stoichiometric ADCs for targeted cancer immunotherapy.

## INTRODUCTION

Antibody–drug conjugates belong to a relatively new class of targeted immunotherapeutics featuring a combination of monoclonal antibody specificity and potency of highly effective cytotoxic agents. ADCs offer selective drug delivery [1], can bypass certain multidrug resistance mechanisms [2], and carry drugs whose toxicity precludes their use as individual antitumor agents [3]. Conventionally, antibody modification has relied on chemical conjugation, wherein activated analogs of cytotoxic agents incorporating biodegradable linkers target the free amino groups of lysine residues or the thiol groups of cysteine residues. Consequently, biologics generated through chemical conjugation exhibit heterogeneity regarding both the payload stoichiometry and the specific conjugation sites. This molecular variability reduces *in vivo* effectiveness [4, 5]. Enzymatic conjugation using microbial transglutaminase overcomes this stoichiometric heterogeneity [4, 5]. Transglutaminases, or protein-glutamine γ-glutamyltransferases, are enzymes catalyzing the cross-linking between the γ-carboxamide groups of glutamine residues and various primary amines. Enzyme recognition and selectivity are governed by a specific amino acid environment and steric accessibility[5]. ADCs are produced using microbial transglutaminase (mTGase), a bacterial enzyme from *Streptomyces mobaraensis*. Bacterial enzymes are cofactor-independent and exhibit high catalytic stability across a broad range of conditions [6]. Although natural antibodies contain several dozen glutamine residues, microbial transglutaminase catalyzes transamidation at a single site: Q295 in the constant region of the IgG heavy chain. In a native molecule, this amino acid residue is shielded by N-glycan; this steric hindrance needs to be overcome to ensure conjugation. The first successful solution to this problem was achieved through using amidase PNGase F [7]. One of the challenges of utilizing mTGase in solution is the concomitant generation of undesired side products, such as mTGase homodimers and aggregates, as well as mTGase–antibody conjugates and antibody conjugates bearing deamidated glutamine fractions [5]. Transglutaminase immobilization on a solid support offers greater control over the reaction stoichiometry [8], effectively suppressing these side reactions.

This study assessed the efficiency of various approaches aimed at immobilizing mTGase on different supports through different strategies, such as (i) the formation of a non-covalent complex, (ii) classical bioconjugation with an epoxy-activated support, and (iii) self-immobilization of mTGase on a support containing free amino groups. The conditions of the immobilization reaction were optimized, and the conditions were established for the most efficient production of the model ADC based on clinically relevant antibody trastuzumab.

## EXPERIMENTAL

**mTGase immobilization**

mTGase (Biopreparat, Russia) was used in the experiment. The protein-derived product was lyophilizate of mTGase from *S. mobaraensis* with dextran; the initial mixture contained 1% enzyme. The mTGase–dextran mixture was separated on a HiTrap desalting chromatography column (Cytiva, USA).

Dynabeads M-280 (Thermo Scientific, USA) and MagStrep XT (CubeTech, Germany), magnetic particles, as well as the Strep-Tactin Sepharose chromatography sorbent (IBA Lifesciences, Germany), were used for mTGase immobilization by high-affinity streptavidin–biotin interaction. mTGase was biotinylated using EZ-Link Sulfo-NHS-LC-biotin (Thermo Scientific, USA). For binding, the biotinylated protein and the Strep-Tactin-containing sorbent were incubated in a buffer solution (50 mM potassium phosphate, 100 mM sodium chloride, pH 6.5). The mTGase concentration was chosen so as to ensure an excess of protein based on the substrate capacity specified by the manufacturer (samples 1–3).

Bioconjugation to epoxy-activated agarose was used as another universal protein immobilization strategy. The mTGase concentration for providing an excess of protein was chosen based on the capacity of a resin containing 90 µmol epoxy groups per mL. The enzyme was immobilized in a 100 mM potassium bicarbonate solution, pH 9.5, at 30°C under rotary agitation for 24 h. An excessive amount of ethanolamine (up to 200 mM) was then added, and the mixture was incubated under stirring. The prepared sorbent with the protein immobilized on it was washed with a 100 mM potassium bicarbonate solution, deionized water, and PBS (sample 4). Simultaneously, we tested whether the conjugation was feasible in a solution containing 50 mM potassium phosphate, 100 mM sodium chloride, and 2 mM dithiothreitol, pH 7.5 (sample 5).

AH-Sepharose 4B (Cytiva, Sweden) and the Unigel-65 amine-modified polymethacrylate matrix were used as sorbents for mTGase self-immobilization. For conjugation, the concentrated enzyme was incubated in a buffer solution (50 mM potassium phosphate, 100 mM sodium chloride, 2 mM dithiothreitol, pH 6.5) under stirring for 24 h at room temperature. For samples 6 and 7, preparation of enzyme samples for self-immobilization on the AH-Sepharose 4B and Unigel-65 resins did not involve desalting on the HiTrap chromatography column; the dextran–mTGase mixture was dissolved in the minimum possible volume of the buffer solution.

**Evaluation of the activity of immobilized mTGase using a low-molecular-weight model substrate**

The sorbent was suspended in PBS (pH 7.4) at a 1 : 1 (vol.) ratio. Either 2 µL of the suspension and 8 µL of PBS samples (samples 1–3) or 5 µL of the suspension and 5 µL of PBS samples (samples 4–13) was transferred to a 500 µL microcentrifuge tube. The substrate solution (40 µL) in an aqueous buffer (0.067 M Cbz-Gln-Gly-OH, 0.22 M NH_2_OH·HCl, 0.44 M Tris, pH 6.0) was added, and the mixture was incubated at 37°C under stirring for 10 min. The reaction was stopped by adding 50 µL of a 0.1 M aqueous FeCl_3_ solution supplemented with 0.3 M CCl_3_COOH and 0.8 M HCl. The resulting solution was dissolved with water to bring the volume to 500 µL, and absorbance at 525 nm was measured. The amount of the enzyme catalyzing the conversion of 1 µmol Cbz-Gln-Gly-OH to hydroxamate per min was taken as one unit of mTGase activity. The specific activity of the immobilized enzyme was calculated using the formula:

*Activity* (U/mL) = (*A*_525_ × *V*)/(ε × *l* × *t* × *v*) = *A*_525_/*v* × 0.147,

where *A*_525_ is the absorbance, *V* is the cuvette volume (500 µL), ε is the molar extinction coefficient (340 L·mol^-1^·cm^-1^), *l* is the optical path length of the cuvette (1 cm), *t* is the reaction time (10 min), and *v* is the sorbent volume (mL).

**Evaluation of the activity of immobilized mTGase in the transamidation reaction of deglycosylated trastuzumab**

The aminotriazide solution in dimethyl sulfoxide (2.7 µL, 100 mM, 270 nmol, 390 eq) and 100 µg of deglycosylated trastuzumab in PBS (4.3 µL, 23.4 mg/mL, 0.69 nmol, 1 eq) were added to the suspension of the immobilized enzyme in 50 µL of PBS; the mixture was incubated at 23°C under stirring for 20 h. The extent of reaction completion was determined by electrophoretic separation under reducing conditions.

## RESULTS AND DISCUSSION

mTGase was immobilized on solid supports using strategies based on three different principles (*Fig. 1*). The interaction between the biotinylated enzyme and streptavidin-modified particles (samples 1–3 in *Table 1*) was selected so as to obtain a non-covalent complex. Conjugation to epoxy-activated agarose (samples 4 and 5 in *Table 1*) was considered a universal method for covalent immobilization of proteins. The feasibility of mTGase self-immobilization on sorbents functionalized with terminal free amino groups (samples 6–11 in *Table 1*) was evaluated based on the substrate specificity of the reaction.

**Fig. 1:**
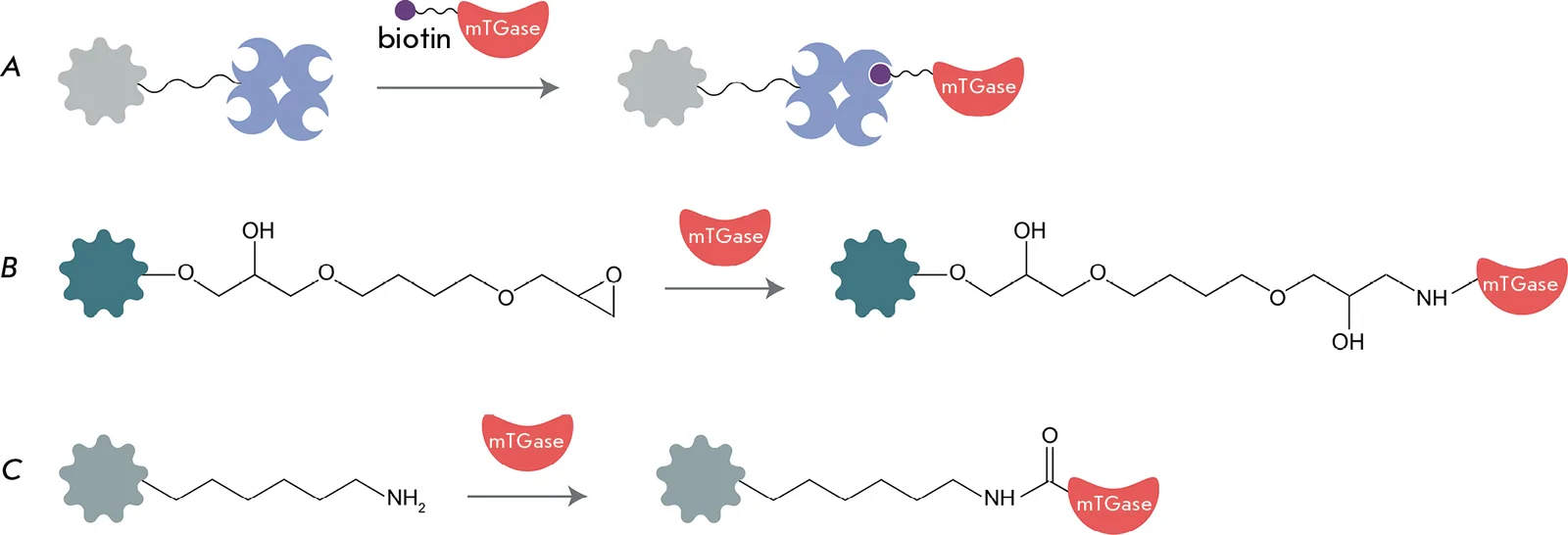
Schematic representation of mTGase immobilization on solid supports. (*A*) Binding of streptavidin-modified particles to the biotinylated enzyme; (*B*) covalent immobilization on epoxy-activated agarose; (*C*) self-immobilization on a sorbent containing free amino groups (AH-Sepharose 4B as an example)

**Table 1:** Conditions of mTGase immobilization on solid supports

Sample	Solid phase (support)	Immobilized enzyme	mTGase concentration	pH of the solution upon binding
1	Dynabeads M-280	Biotinylated mTGase	30 µg/mL	6.5
2	MagStrep XT	Biotinylated mTGase	50 µg/mL	6.5
3	Strep-Tactin Sepharose	Biotinylated mTGase	15 mg/mL	6.5
4	Epoxy-activated agarose	mTGase	10 mg/mL	9.5
5	Epoxy-activated agarose	mTGase	10 mg/mL	7.5
6	AH-Sepharose 4B	1% mTGase in dextran	3 mg/mL	6.5
7	Unigel-65	1% mTGase in dextran	3 mg/mL	6.5
8	AH-Sepharose 4B	mTGase	3 mg/mL	6.5
9	Unigel-65	mTGase	3 mg/mL	6.5
10	AH-Sepharose 4B	mTGase	12 mg/mL	6.5
11	Unigel-65	mTGase	12 mg/mL	6.5

The activity of immobilized mTGase was measured using the standard procedure [9, 10]. Sample activity is shown in *Fig. 2*.

**Fig. 2:**
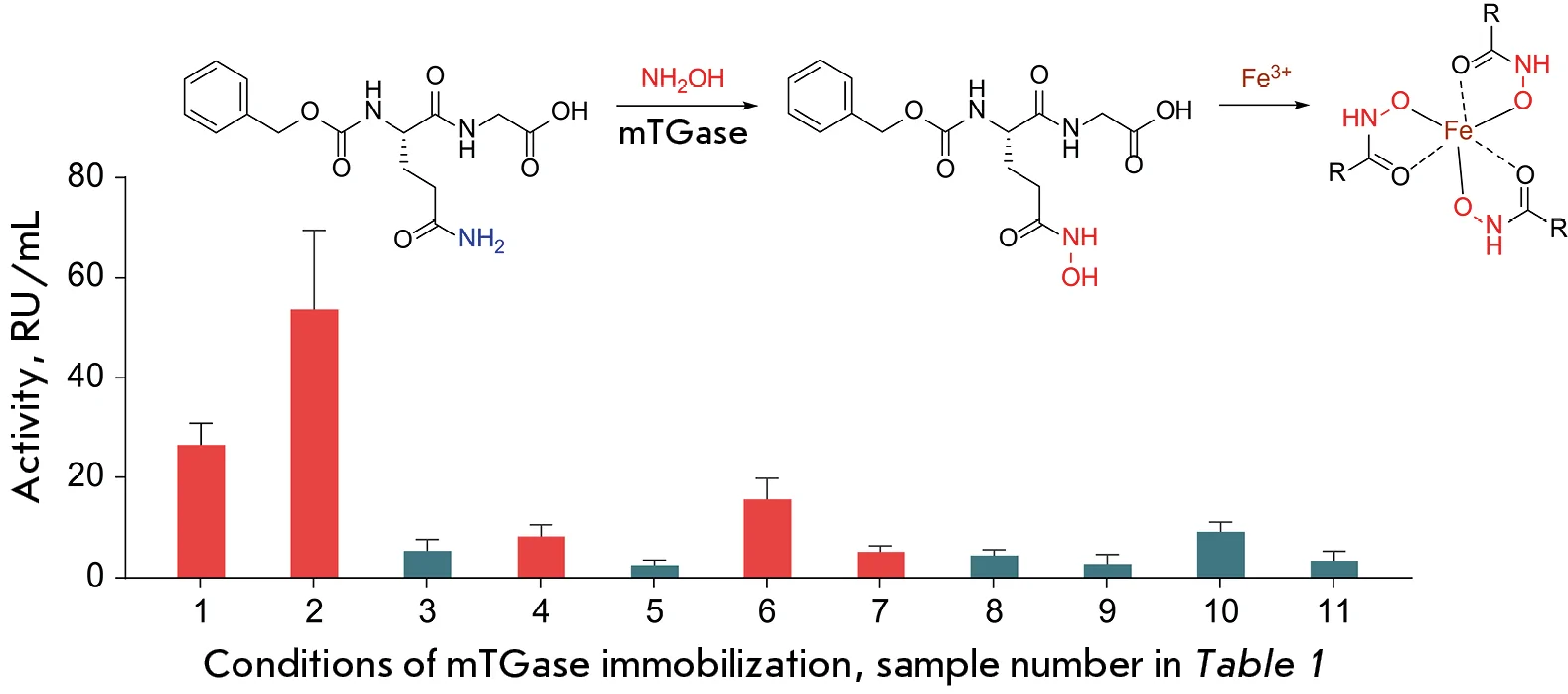
Schematic representation of the model reaction and the specific activity of immobilized mTGase. Samples with activity in the trastuzumab transamidation reaction shown in the representative electropherogram in *Fig. 3* are highlighted in red on the bar graph. Data are presented as a mean ± standard deviation (*n* = 3)

Immobilization on MagStrep XT magnetic particles (53.7 ± 15.9 RU/mL, sample 2) yielded the highest specific activity upon hydroxamate formation; a twice-lower level of activity was observed for the enzyme bound to Dynabeads M-280 magnetic particles (26.5 ± 4.5 RU/mL, sample 1). The highest specific activity of mTGase immobilized via transamidation with the amino groups of the AH-Sepharose 4B (sample 6) and Unigel-65 (sample 7) sorbents and bioconjugation to epoxy-activated agarose (sample 4) was 15.8 ± 4.0, 5.1 ± 1.3, and 8.2 ± 2.2 RU/mL, respectively. For self-immobilization, the maximum values were achieved when using the crude enzyme preparation in the dextran matrix without any intervening purification steps.

Activity of the immobilized enzyme was then assessed in the reaction modeling its real application to produce the ADC. Trastuzumab, a targeted monoclonal antibody used in clinical practice to treat breast cancer both as monotherapy [11] and as a component of conjugates [12, 13], was selected as the targeting platform. Trastuzumab is a recombinant, humanized IgG1 antibody. It specifically binds to human epidermal growth factor receptor 2 (HER2), whose overexpression correlates with the highly aggressive phenotypes of breast carcinoma [13].

Testing the activity of immobilized mTGase involved transamidation of deglycosylated trastuzumab at the Gln295 residue and insertion of a triazide linker described previously [14] (*Fig. 3*). We have demonstrated earlier [14] that this branched linker ensures a final drug-to-antibody ratio (DAR) of 6 and enhances drug effectiveness compared to that of unbranched linkers (DAR = 2).

**Fig. 3:**
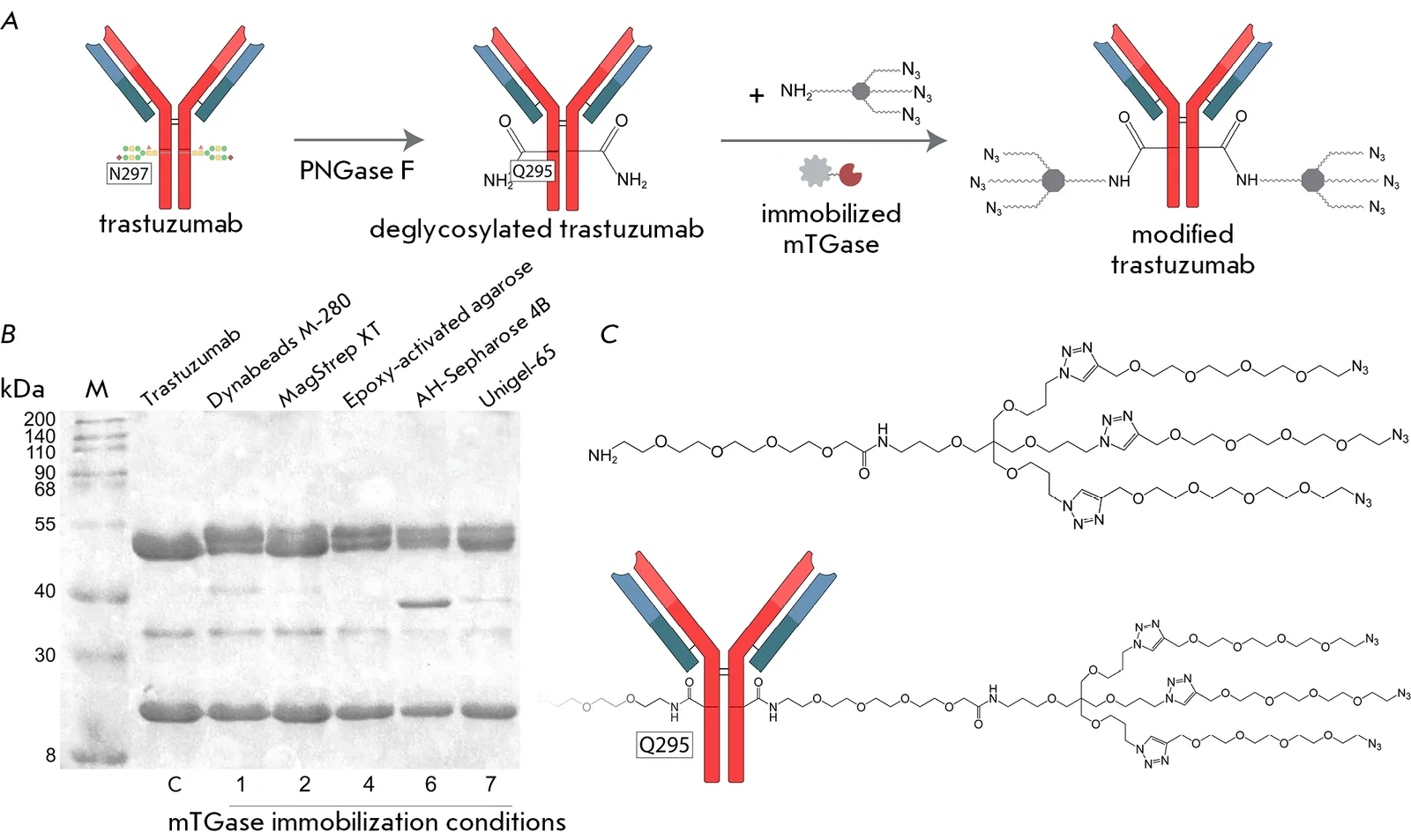
(*A*) Schematic representation of the site-specific modification of trastuzumab at Q295; (*B*) representative electropherogram (15% PAGE). Unmodified deglycosylated trastuzumab is applied as a negative control; (*C*) the structures of the aminotriazide used for modification and the conjugate product

The highest macromolecular conversion efficiency was observed for the binding of biotinylated mTGase to Dynabeads M-280 magnetic particles. The degrees of conversion for immobilization on epoxy-activated agarose and self-immobilization upon interaction with the primary amines of the AH-Sepharose 4B sorbent were almost identical (~50%). However, the conditions of immobilization and sorbent washing to minimize enzyme leaching need additional optimization in the case of the AH-Sepharose 4B sorbent. The Unigel-65 matrix displayed poor bioconjugation performance, mirroring its low activity toward small-molecule substrates; this limitation is presumably related to a shorter spacing linker and heightened steric hindrance constraining substrate accessibility to the bound enzyme. A sharp inconsistency was observed for the MagStrep XT streptavidin magnetic particles: despite displaying the highest specific activity during initial colorimetric screening with the low-molecular-weight substrate Cbz-Gln-Gly-OH, they demonstrated low conversion efficiency during antibody modification. This drop in performance is with a high probability indicative of restricted steric accessibility when accommodating a high-molecular-weight macromolecular substrate like a full-length IgG antibody within the restricted surface microenvironment of the solid support.

## CONCLUSION

This study has evaluated three distinct operational strategies of microbial transglutaminase immobilization to facilitate site-specific antibody modification: streptavidin–biotin interaction, covalent attachment to an epoxy-activated sorbent, and self-immobilization on amine-functionalized matrices. Although each of these approaches enables immobilization of the active enzyme, the application of Dynabeads M-280 streptavidin-modified magnetic particles and biotinylated mTGase ensured the highest conversion degree of the model monoclonal antibody trastuzumab. MTGase immobilized via epoxy-activated agarose and a AH-Sepharose 4B amine-modified sorbent exhibited reduced catalytic performance toward both the low-molecular-weight substrate and full-length antibody target. However, these covalent and amine-directed approaches present superior scalability, rendering them better suited for preparative production of ADCs. Although the self-immobilization strategy offers a highly cost-effective paradigm, it demands further parameter optimization to unleash its operational potential. These insights offer novel approaches to optimizing enzymatic bioconjugation workflows and advancing the scalable production of stoichiometrically defined immunotherapeutics with enhanced *in vivo* efficacy profiles.
